# Ultraintense UV emission from ZnO-sheathed ZnS nanorods

**DOI:** 10.1038/s41598-017-13556-0

**Published:** 2017-10-12

**Authors:** Jae Kyung Lee, Gun-Joo Sun, Woo Seok Lee, Soong Keun Hyun, Kyoung-Kook Kim, Seung-Bok Choi, Chongmu Lee

**Affiliations:** 1 Department of Materials Science and Engineering, Inha University, Incheon, 402-751 Republic of Korea; 2 Department of Nano-Optical Engineering, Korea Polytechnic University, 2121 Jeongwangdong, Shiheung City, Gyeonggido 429-793 Republic of Korea; 3 Department of Mechanical Engineering, Inha University, Incheon, 402-751 Republic of Korea

## Abstract

Short-wavelength luminescence is essential for high-performance optoelectronic device applications. There have been efforts to obtain intense ultraviolet (UV) emission by encapsulating ZnO one-dimensional (1D) nanostructures with materials such as ZnS. However, the encapsulation of ZnS 1D nanostructures with ZnO has not been reported. In this paper, we report ultraintense UV emission from ZnS nanorods coated with ZnO, *i.e*., ZnS-core/ZnO-shell nanorods. UV emission from the ZnS-core/ZnO-shell nanorods was much more intense than that obtained from the extensively studied ZnO-core/ZnS-shell nanorods. The highest intensity of the near-band-edge emission from the ZnS-core/ZnO-shell nanorods was obtained with a ZnO shell layer thickness of 35 nm, which is ∼16 times higher than that of pristine ZnS nanorods. Moreover, the deep level (DL) emission was suppressed completely. The substantial enhancement of the UV emission from the ZnS nanorods and the complete suppression of the DL emission by ZnO sheathing can be rationalized by combining the following four effects: the reinforcement of the UV emission by the overlap of the UV emissions from the ZnS core and ZnO shell, enhancement of the emission from the ZnO shell by the carrier transfer from the ZnS core to the ZnO shell, suppression of the capture of carriers by the surface states on the ZnS surface, and suppression of the visible emission and nonradiative recombination in ZnS.

## Introduction

Short-wavelength luminescence is essential for high-performance optoelectronic devices such as white light emitting diodes (LED), compact disks/digital video disks with high information storage capacities, and short-wavelength laser diodes (LDs) for next generation optical communication. 2–6 compound semiconductors such as ZnO, ZnS, ZnSe, and ZnTe as well as the 3–5 compound semiconductor GaN are promising short-wavelength light emitting materials^1,2^. In particular, of the four 2–6 compound semiconductors, ZnO and ZnS have been studied extensively, over the past few decades.

ZnO presents many merits such as a direct wide band gap of 3.37 eV and low power threshold for optical pumping. ZnO also has many advantages over GaN, such as a large exciton binding energy (60 meV), thermal and chemical stabilities in air, low epitaxial growth temperature, excellent radiation resistance, and the availability of ZnO substrates^3^. In achieving short-wavelength light emission, it is essential to enhance the near-band-edge (NBE) ultraviolet emission and suppress the deep level (DL) green emission from the ZnO nanostructures. Over the past decades, various techniques, including thermal annealing, plasma treatment, doping, decoration with nanoparticles, and core-shell structure formation, have been studied for this purpose^4–6^. Of these techniques, encapsulation of ZnO 1D nanostructures with other materials has been studied most widely. Various materials, including ceramics (ZnS, SnO_2_, Al_2_O_3_, MgO, and ZnCdO), metals (Zn, Au, Ag, and Pt), and polymers (polyaniline^7^ and poly-methyl methacrylate), have been investigated as sheath materials.

On the other hand, ZnS is also a short-wavelength light-emitting material with a direct band gap of 3.66 eV. ZnS has many applications in the fields of flat-panel displays^8^, sensors, lasers^9,10^, and photodetectors^11^. Compared to ZnO, ZnS has attracted less attention because of its higher density of defects. The defect states in ZnS nanostructures, such as surface states, stoichiometric vacancies, and interstitial lattice defects cause unstable optical and electrical properties, as well as poor reliability of the ZnS-based optoelectronic devices. Owing to these defects, the PL intensity of the ZnS nanostructures is known to deteriorate over a period of few days, if they are not annealed^12^.

There have been many efforts to enhance the NBE emission from ZnO 1D nanostructures and suppress their DL emission by sheathing them with ZnS thin films. Despite these efforts, the intensity of the NBE emission was not enhanced significantly, as seen in Table 1 ^13–15^. Among these efforts, the best results were obtained by Li *et al*.^13^ The intensity of the NBE emission of ZnO nanowires increased ~5 times, and the DL emission was suppressed completely upon sheathing them with ZnS thin films. It was widely believed that the ZnO 1D nanostructures had to be coated with materials such as ZnS to achieve intense, stable, and reliable UV emission. However, no attempts to sheath the ZnS 1D nanostructures with ZnO have been made, possibly because the ZnS nanostructures have a higher density of defects than ZnO nanostructures. In this study, ZnS-core/ZnO-shell 1D nanostructures were prepared, and their photoluminescence properties were examined for the first time, to the best of our knowledge. The NBE emission intensity of the ZnS nanorods increased by more than 16 times, and the DL emission was suppressed completely after sheathing with ZnO, which is much better than that obtained by sheathing the ZnO nanorods with ZnS and those of most of the high quality ZnO nanostructures reported up to date as shown in Table 2 ^16–21^.

**Table 1:** Comparison of the NBE emission intensity ratios of the ZnO-core/ZnS-shell or ZnS-core/ZnO-shell 1D nanostructures with those of pristine ZnO nanostructures.

Core material^*^	Shell material	I_NBE_/I_0_	Comments	Reference
ZnS NWs	ZnO	16.3	I_DL_ = ~0	Present study
ZnO NTs	ZnS	5.29	I_DL_ = ~0	13
ZnO NPs	ZnS	1.35	DL emission exists. I_NBE_/I_DL_ = ~11	14
ZnO NRs	ZnS	1.15	DL emission exists. I_DL_ > I_NBE_	15

^*^NWs: nanowires, NTs: nanotubes, NPs: nanoparticles, and NRs: nanorods.

**Table 2:** Comparison of the intensity ratio of the NBE emission to the DL emission, *I*_*NBE*_ */I*_*DL*_ of the ZnS-ZnO core-shell nanorods with those of other high quality ZnO nanomaterials.

Type of ZnO nanostructures	*I*_*NBE*_ */I*_*DL*_	Comments	Reference
ZnS-ZnO core-shell nanorods	~1,000		This study
ZnO nanowalls	1.5	Aqueous chemical growth below 100 C	16
ZnO nanorods	33.3	Aqueous chemical growth below 100 C	16
ZnO nanoflowers	5.8	Aqueous chemical growth below 100 C	16
ZnO nanotubes	10.7	Aqueous chemical growth below 100 C	16
ZnO nanowires	24.9	Hexagonal-shaped	17
ZnO nanosaws	16.8	Thermal evaporation at 460 C/annealed at 700 C	18
ZnO thin films	10.0	PLD at 400 C	19
ZnO thin films	~1,000	MOCVD at 65 Pa	20
Hierachical ZnO structures	28.3		21

## Results

**Figure 1:**
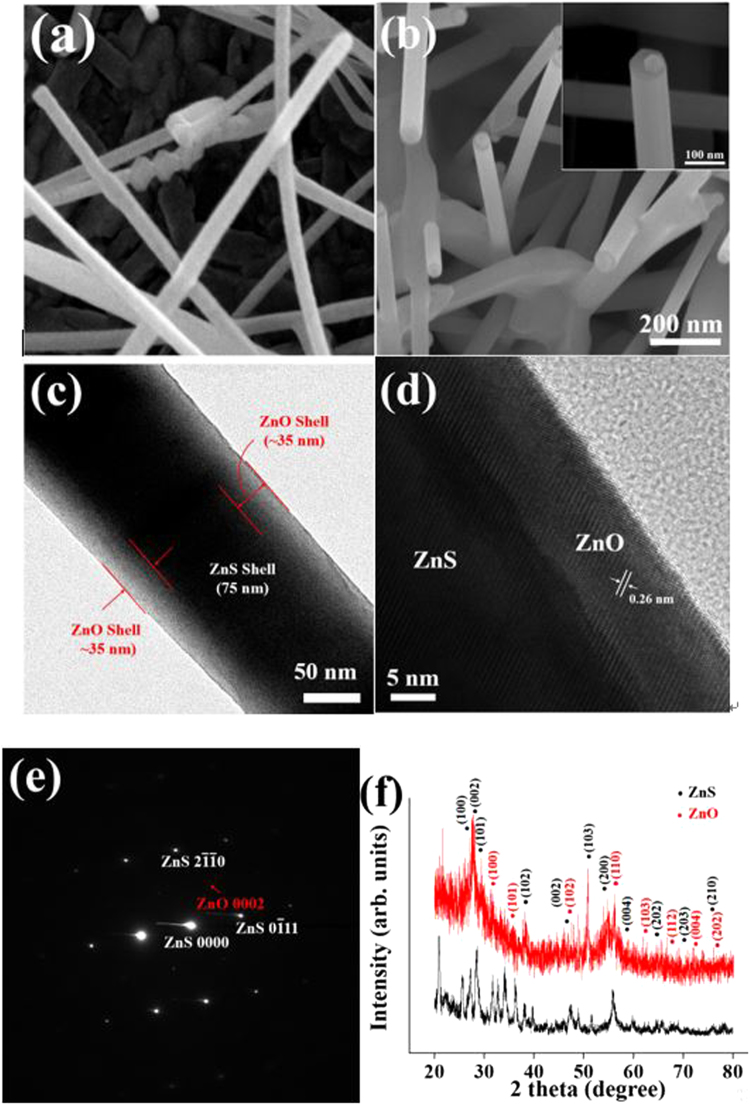
(**a**) SEM image of pristine ZnS nanorods. (**b**) SEM image of ZnO-sheathed ZnS nanorods. Inset, ZnO-sheathed ZnS nanorod with a gold nanoparticle on its tip. (**c**) Low-magnification TEM image of a typical ZnS-core/ZnO-shell nanorod. (**d**) HRTEM image of the ZnS-ZnO interface. (**e**) The corresponding SAED pattern. (**f**) XRD patterns of pristine ZnS and ZnO-sheathed ZnS nanorods.

Figure 1 and b show the scanning electron microscopy (SEM) images of pristine ZnS nanorods and ZnO-sheathed ZnS nanorods, *i.e*., ZnS-core/ZnO-shell nanorods, respectively. The morphology of the core-shell nanorods is similar to that of pristine nanorods. However, the mean diameter of the former appears larger than that of the latter. The number of atomic layer deposition (ALD) cycles used for the ZnO shell formation was 250. The diameter of the nanorods ranged from 40 to 160 nm. A gold nanoparticle is observed at the tip of each nanorod, suggesting that the ZnS nanorods were grown by the vapor-liquid-solid (VLS) mechanism^22^. The tips of all the pristine nanorods and most core-shell nanorods are spherical, as shown in the inset of Fig. 1a. Hexagonal-shaped core-shell nanorods are seldom observed (inset of Fig. 1b). The hexagonal shape is considered to be the characteristic of ZnO rather than ZnS, and hexagonal ZnO rods have been reported frequently^23^. The length of the nanorods ranged from 1 to 2 μm. The SEM image in Fig. 1c shows that the nanorod has a core-shell structure. The shell region is distinguished from that of the core by the difference in the contrast. The ZnO shell appears brighter than the ZnS core, because ZnO has much higher transmittance than ZnS. A line is drawn as a guide to the eye in Fig. 1c to distinguish the core and shell regions. The diameter of the core and thickness of the shell layer are ~75 and ~35 nm, respectively, for 250 cycles of ALD.

**Figure 2:**
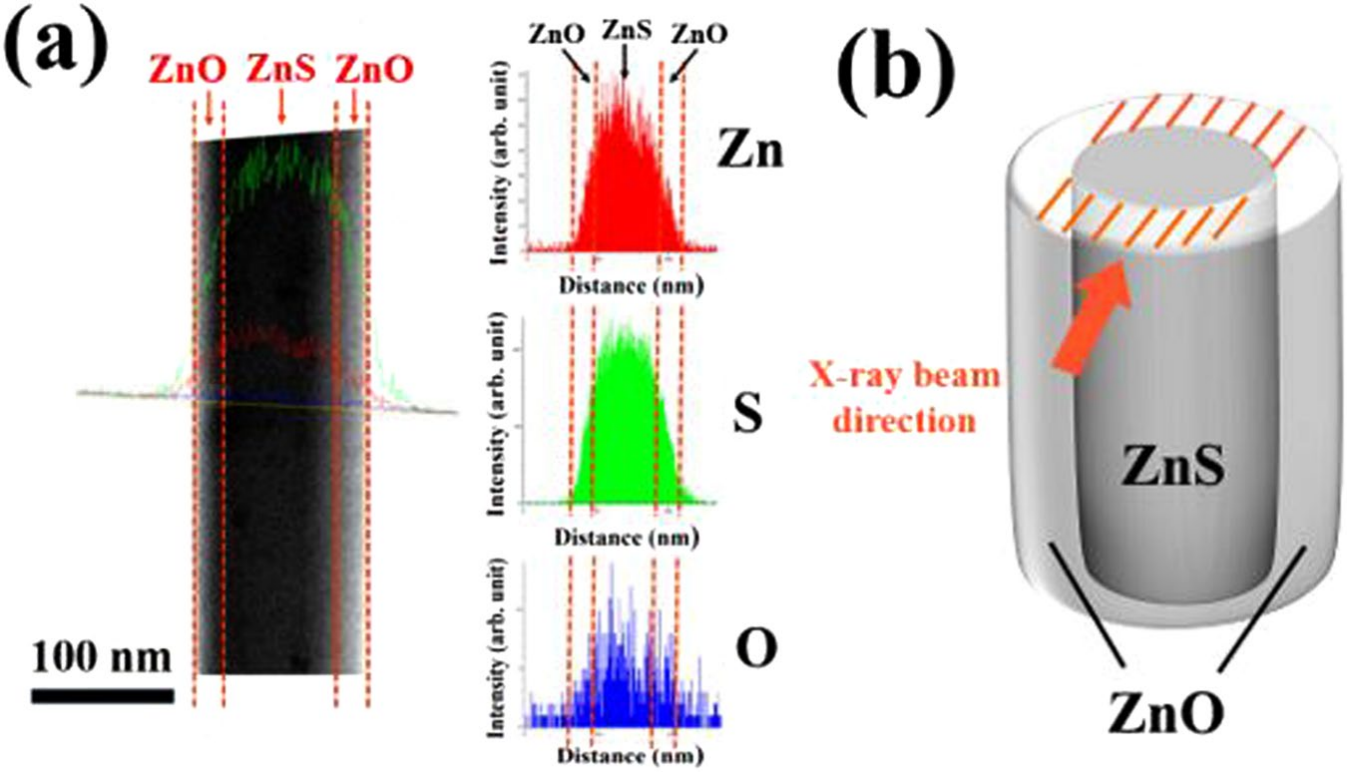
(**a**) EDS line-scanning elemental concentration profile of a typical ZnS-core/ZnO-shell nanorod. (**b**) Schematic showing the overlapping of two ZnO shell layers and a ZnO core layer in a typical ZnS-core/ZnO-shell nanorod along the incident X-ray beam direction.

Figure 1d shows a high-resolution transmission electron microscopy (HRTEM) image of the interface of the ZnS-core/ZnO-shell structure. The corresponding selected area electron diffraction (SAED) pattern is shown in Fig. 1e. The clear spots forming the rectangular lattice are reflections from the ZnS core, whereas the dim spots are from the ZnO shell. The spotty patterns in the SAED image reveal that both the ZnS core and the ZnO shell are monocrystalline. Figure 1f presents the XRD pattern of the ZnS-core/ZnO-shell nanorods obtained using the Cu-Kα radiation (λ = 0.1541 nm). The diffraction pattern of pristine ZnS nanorods confirms that the crystal structure corresponds to the standard wurtzite ZnS (JCPDS card No. 89–2942). All the sharp diffraction peaks in the pattern could be indexed to the wurtzite structure. The lattice constants derived from the peak positions are *a* = 0.3818 nm and *c* = 0.6260 nm, which are in good agreement with those of bulk ZnS crystals. On the other hand, a few reflection peaks assigned to the wurtzite ZnO phase (JCPDS card No. 89–1397) were observed in addition to the wurtzite ZnS phase, in the XRD pattern of the ZnS-ZnO core-shell nanorods. The line scanning energy dispersive X-ray spectroscopy (EDS) elemental concentration profiles of the core-shell nanorods are shown in Fig. 2a. The results confirm the ZnS-ZnO core-shell nanostructures even though the EDS elemental concentration profiles still seem to show some sulfur in the shell regions maybe because of the inaccuracy of the EDS technique. A close examination of the distributions of sulfur and oxygen along the diameter of the nanorod confirms ZnS core and ZnO shell structure of the nanorod. The high oxygen concentration at the core region might be due to the overlap of the front and rear ZnO shell layers with the ZnS core in the path of the X-ray beam passing through the core-shell nanorod sample in the EDS measurement (Fig. 2b).

**Figure 3:**
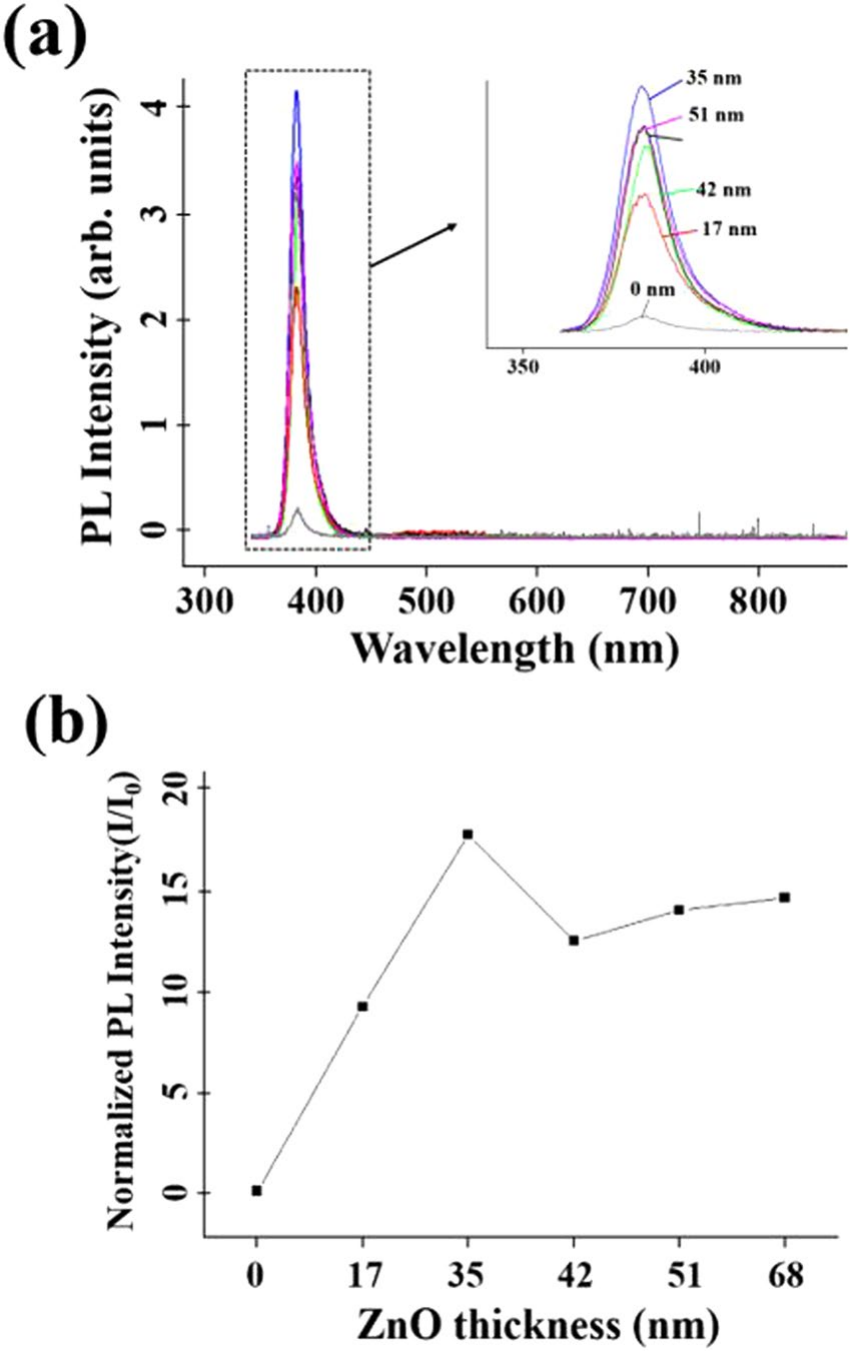
(**a**) PL spectra of ZnS-core/ZnO-shell nanorods with various shell layer thicknesses. (**b**) PL intensity of the core-shell nanorods (I) normalized by that of the pristine ZnS nanorods without the ZnO shell (I_0_) versus the ZnO shell thickness.

Figure 3a displays the room-temperature photoluminescence (PL) spectra of the pristine ZnS nanorods and ZnS-core/ZnO-shell nanorods with different shell layer thicknesses. The pristine ZnS nanorods (without the ZnO shell) show a relatively weak UV emission band at ∼382 nm corresponding to the NBE emission, along with a far weaker and broad DL emission band centered at ∼522 nm. The NBE emission intensity of the core-shell nanorods exhibits strong dependence on the ZnO shell layer thickness. The highest intensity of the NBE emission from the ZnS-core/ZnO-shell nanorods was obtained for a ZnO shell thickness of 35 nm. The NBE emission intensity of the nanorods for a shell thickness of 35 nm is ∼16 times higher than that of the pristine nanorods (Fig. 3b). Moreover, the DL emission was suppressed completely. A high ratio of the NBE emission to the DL emission is essential for realizing high quality UV optoelectronic devices such as LEDs and LDs.

## Discussion

The NBE emission from ZnS or ZnO nanostructures is associated with the excitons bound to shallow donors, whereas the DL emission is associated with the oxygen vacancy-related defects^24,25^ such as singly-ionized oxygen vacancies, which can easily form recombination centers. The substantial enhancement of the NBE emission from the ZnS nanorods enabled by the ZnO sheathing can be rationalized by combining the following four effects.

First, the UV emission is reinforced by the overlap of the NBE emissions from the ZnS core and ZnO shell because the wavelength range of the UV emission from ZnO overlaps that from ZnS. According to previously published studies^26–30^, the NBE emission peak from ZnO appears over the wavelength range of 361–393 nm. On the other hand, the NBE emission peak from ZnS appears over 340–400 nm, depending on the morphology, synthetic method, and synthesis temperature of the ZnS nanostructures (see Table 3 for the comparison)^31–34^. Accordingly, it is not simple to deconvolute the two UV emissions, *i.e*., the UV emission from ZnO and that from ZnS, because the former and the latter overlap with each other.

**Table 3:** Comparison of the wavelengths of the NBE emission peaks in the room temperature PL spectra of various ZnS 1D nanostructures.

ZnS nanostructures	Synthesis method	λ of NBE emission peak (nm)	Reference
Nanobowls	self-assembled monolayer polystyrene sphere template floating on a precursor solution (a mixture of zinc acetate solution, ammonium acetate, disodium ethylenediamine tetraacetic acid and thioacetamide)	382	^33^
Nanowires	thermal evaporation of ZnS powder in the presence of an Au catalyst	398	^34^
Nanoribbons	thermal evaporation of ZnS powder in the presence of an Au catalyst	398	^34^
Nanoribbons	CVD of Zn and S powders in the different temperature zones	390	^35^
Nanoparticles (bulk)	Dispersion and continuum sonication of ZnS powder dispersed in isopropyl alcohol	348	^36^
Nanoparticles (nanoparticles)	Dispersion and continuum sonication of ZnS powder dispersed in isopropyl alcohol	374	^36^

**Figure 4:**
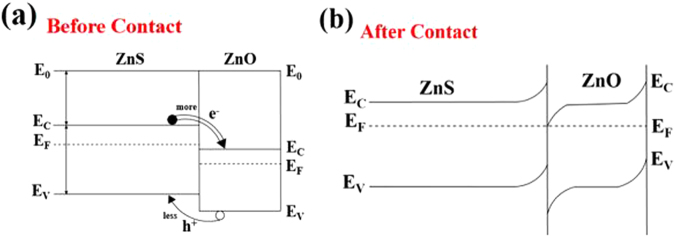
Energy band diagrams of the ZnS-ZnO couple: (**a**) Before contact and (**b**) after contact.

**Figure 5:**
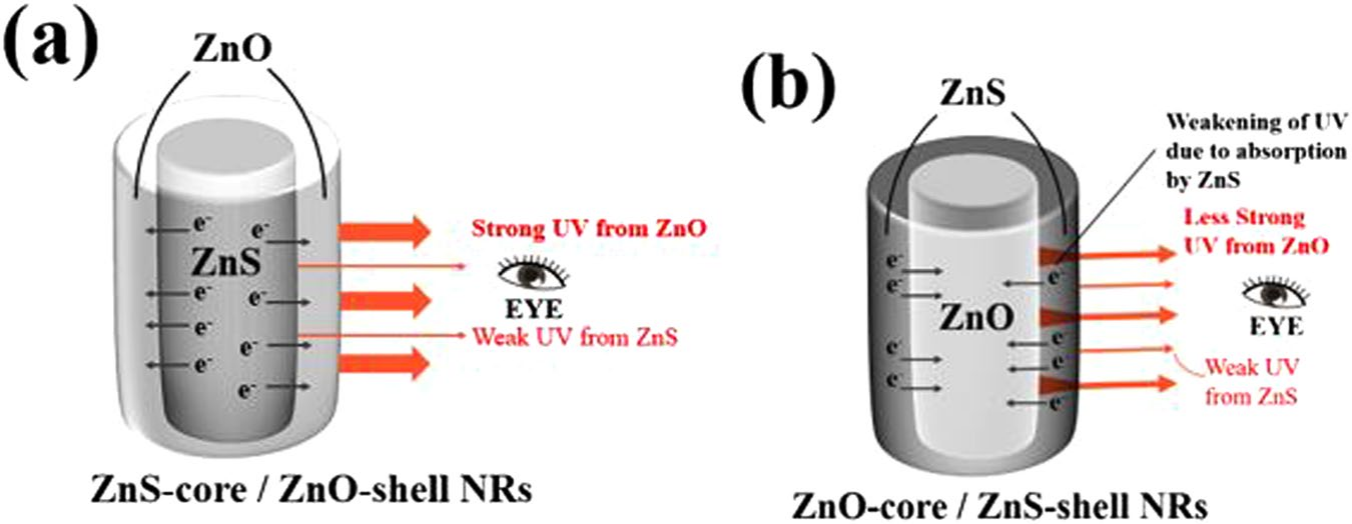
Resolved UV emission from (**a**) ZnS-core/ZnO-shell and (**b**) ZnO-core/ZnS-shell nanorods with respect to the source material.

Second, the emission from the ZnO shell is enhanced by the carrier transfer from the ZnS core to the ZnO shell. When the ZnS-core/ZnO-shell nanorods are excited by the He-Cd laser, electron-hole pairs, *i.e*., photoelectrons and photoholes are generated in both the ZnS core and ZnO shell. Figure 4a and b present the energy band diagrams of the ZnS-ZnO couple before and after contact. The electrons in the ZnS core tend to transfer to the ZnO shell owing to its higher *E*_*C*_ than that of ZnO, whereas the holes in the ZnO shell tend to transfer to the ZnS core owing to the lower *E*_*V*_ of ZnO than that of ZnS (Fig. 4a). The number of electrons transferred from ZnS to ZnO is larger than the number of holes transferred in the reverse direction, because the *E*_*F*_ of ZnS is higher than that of ZnO. Accordingly, the carrier density in the ZnO shell is higher than that in the ZnS core. After the transfer of the electrons and holes, electron-hole recombination occurs in both the ZnS core and the ZnO shell, to generate photons. The recombination probability is higher and therefore more photons are produced in the ZnO shell than in the ZnS core. Therefore, the emission from the ZnS core is weaker than that from the ZnO shell. Furthermore, the weak UV light emitted from the ZnS core is partly absorbed by the ZnO shell layer before it reaches our eyes, even though the re-absorption is not significant because the refractive index of ZnO is not high (*n*_ZnS_ = 2.47^35^, *n*_ZnO_ = 1.93^36^). In contrast, the more intense UV emission from the ZnO shell is seldom reabsorbed by the ZnS core layer before it reaches our eyes, as shown in Fig. 5a. Therefore, the UV emission from the ZnS-core/ZnO-shell nanorods is a combination of the weak UV emission from the ZnS core and the strong UV emission from the ZnO shell.

In the ZnO-core/ZnS-shell, the strong UV light emitted from the ZnO core is weakened while it passes through the ZnS shell owing to its absorption by the ZnS (Fig. 5b). Consequently, the UV emission intensity is significantly lowered. On the other hand, the UV light emitted from the ZnS shell is not absorbed by the ZnO core before it reaches our eyes, but its intensity is initially low because of the transfer of the electrons from the ZnS shell to ZnO core. Therefore, the UV emission from the ZnO-core/ZnS-shell nanorods is a combination of the weak UV emission from the ZnO core and less strong UV emission from the ZnS shell. In summary, UV light with a higher intensity is obtained from the ZnS-core/ZnO-shell nanorods than from ZnO-core/ZnS-shell nanorods.

Third, the capture of the carriers by the ZnS surface states is suppressed. ZnS nanostructures are known to have a higher density of surface states than ZnO. Unless the surface of the ZnS nanorod is passivated, the photogenerated carriers would be readily captured by the surface states, because of the high specific surface area of the nanorods and the short relaxation times for carriers tunneling from the interior of the nanorod to its surface. Consequently, the NBE emission would be quenched in pristine ZnS nanorods^37^. In contrast, the surface state density at the ZnS-ZnO interface of the ZnS-core/ZnO-shell nanorods is relatively low because the surfaces of the ZnS nanorods are well passivated by the ZnO shell layer.

Fourth, the visible emission and nonradiative recombination is suppressed; a depletion region is formed in the outer region of the ZnS core, close to the core-shell interface owing to the electron transfer from the ZnS core to the ZnO shell. The transition process for the visible emission and nonradiative recombination may be suppressed in the depletion region of the ZnS core, where the Fermi energy level is lower than the energy levels of the visible emission-related defects (S vacancies and Zn interstitials) and the nonradiative transition-related defects^38,39^.

## Methods

ZnS rods were synthesized by the thermal evaporation of ZnS powders on 3-nm-thick Au-coated Si (100) substrates for 1 h, using a two-heating-zone horizontal tube furnace^9^. An alumina boat containing the Zn powder used as the precursor and the Si substrate were placed in the first (1,000 °C) and second heating zones (850 °C), respectively, of the tube furnace. The nitrogen gas pressure and flow rate in the chamber were maintained at 0.5 Torr and 500 standard cubic centimeters per minute (sccm), respectively, throughout the synthetic process.

The ZnS nanorods synthesized by thermal evaporation were transferred to an ALD chamber and ZnO thin films were deposited on the ZnS nanorods by ALD. The source gases diethylzinc (DEZn) and H_2_O stored in bubblers at 0 and 10 °C, respectively, were fed alternately into the chamber through separate inlet lines and nozzles. The substrate temperature and pressure in the chamber were maintained at 150 °C and 0.1 Torr, respectively. Typical pulse lengths for DEZn, H_2_O, and the purging of the reactants were 0.15, 0.2, and 3 s, respectively. ZnS-core/ZnO-shell nanorod samples with various shell layer thicknesses were prepared by varying the number of ALD cycles from 0 to 500.

Room-temperature PL was measured using a He-Cd laser (325 nm) as the excitation source. SEM (Hitachi S-4200) was performed to examine the microstructures of the nanorod samples and EDS patterns were obtained on the same SEM system. HR-TEM and SAED (Phillips CM-200, 200 KV) were performed to further examine the phases and microstructures of the samples. Glancing angle (0.5°) XRD (Rigaku DMAX 2500) with Cu-Kα radiation (λ = 0.1541 nm) was performed to identify the phases of the obtained products.
